# SOCIAL CONNECTEDNESS ACROSS THE LIFE COURSE: CORE NETWORKS AND EVERYDAY SOCIAL INTERACTIONS OF AMERICAN ADULTS

**DOI:** 10.1093/geroni/igad104.1840

**Published:** 2023-12-21

**Authors:** Siyun Peng, Adam Roth, Brea Perry

**Affiliations:** Indiana University, Bloomington, Indiana, United States; Oklahoma State University, Stillwater, Oklahoma, United States; Indiana University, Bloomington, Indiana, United States

## Abstract

Scholars have been monitoring the signs of social isolation and weakening of family ties among Americans. This paper asks: (1) Do Americans socially disengage from society as they age? (2) Are family ties replaced by work or friends as Americans grow out of their original family? Using a state representative sample of egocentric network data (Person to Person Study: P2P) and a national representative sample of time dairy data (American Time Use Survey: ATUS) during 2018-2021, we found that although people spent less social time with others as they age, they maintained a stable sized core network of confidants for discussing important and health matters. This refutes the view that old age has a universal negative influence on social connectedness. We found little evidence that family ties are replaced by non-kins at any age in both core network and daily social contact. By combining core network and time dairy data, we found that although people spent substantial time with coworkers daily when they enter the workforce, they rarely included even a single coworker into their core network. This suggests that people’s core network is less influenced by current daily social interactions than many theories on social contact would predict. We speculate that people may organize their core network based on cumulative time they spend with a particular person over the life course rather than current time. The roles of life events and age in shaping changes in daily interactions and core network over the life course are further discussed.

